# Prediction of outcomes in subjects with type 2 diabetes and diabetic foot ulcers in Catalonian primary care centers: a multicenter observational study

**DOI:** 10.1186/s13047-023-00602-6

**Published:** 2023-02-28

**Authors:** Magdalena Bundó, Bogdan Vlacho, Judit Llussà, Isabel Bobé, Meritxell Aivar, Carmen Ciria, Ana Martínez-Sánchez, Jordi Real, Manel Mata-Cases, Xavier Cos, Montserrat Dòria, Jordi Viade, Josep Franch-Nadal, Dídac Mauricio

**Affiliations:** 1 DAP-Cat Group. Unitat de Suport a La Recerca Barcelona Ciutat, Institut Universitari d’Investigació en Atenció Primària Jordi Gol (IDIAP Jordi Gol), 375, Entresuelo. 08025, Barcelona, Spain; 2 Primary Health Care Center Ronda Prim, Gerència d’Àmbit d’Atenció Primària Metropolitana Nord de Barcelona, Institut Català de La Salut, Mataró, Spain; 3 Pharmacology Department, Universitat Autònoma de Barcelona (UAB), Cerdanyola del Vallès, Spain; 4 Institut de Recerca Hospital de La Santa Creu I Sant Pau, Barcelona, Spain; 5 CIBER of Diabetes and Associated Metabolic Diseases (CIBERDEM), Instituto de Salud Carlos III (ISCIII), Barcelona, Spain; 6 Primary Health Care Centre Sant Roc, Gerència d’Àmbit d’Atenció Primària Metropolitana Nord de Barcelona, Institut Català de La Salut, Mataró, Spain Catalan Health Institute, Badalona, Spain; 7 Primary Health Care Center La Mina, Gerència d’Àmbit d’Atenció Primària Barcelona Ciutat, Institut Català de La Salut, Sant Adrià de Besòs, Spain; 8 Primary Health Care Centre Sants, Gerència d’Àmbit d’Atenció Primària Barcelona Ciutat, Institut Català de La Salut, Barcelona, Spain; 9 Primary Health Care Centre Ponts. Gerència d’Àmbit d’Atenció Primària Lleida, Institut Català de La Salut, Lleida, Spain; 10 Primary Health Care Centre El Carmel. Gerència d’Àmbit d’Atenció Primària Barcelona Ciutat, Institut Català de La Salut, Barcelona, Spain; 11 Universitat Internacional de Catalunya, Epidemiologia I Salut Pública, Sant Cugat, Spain; 12 Primary Health Care Center Sant Martí de Provençals, Gerència d’Àmbit d’Atenció Primària Barcelona Ciutat, Institut Català de La Salut, Barcelona, Spain; 13 Innovation office at Institut Català de La Salut, Barcelona, Spain; 14 Department of Endocrinology & Nutrition, Hospital de La Santa Creu I Sant Pau, Barcelona, Spain; 15 Department of Endocrinology & Nutrition, Hospital Germans Trias I Pujol, Badalona, Spain; 16 Primary Health Care Center Raval Sud, Gerència d’Àmbit d’Atenció Primària Barcelona Ciutat, Institut Català de La Salut, Barcelona, Spain; 17 Department of Medicine, University of Vic - Central University of Catalonia, Vic, Spain

## Abstract

**Background:**

Diabetic foot and lower limb complications are an important cause of morbidity and mortality among persons with diabetes mellitus. Very few studies have been carried out in the primary care settings. The main objective was to assess the prognosis of diabetic foot ulcer (DFU) in patients from primary care centers in Catalonia, Spain, during a 12-month follow-up period.

**Methods:**

We included participants with type 2 diabetes and a new DFU between February 2018 and July 2019. We estimated the incidence of mortality, amputations, recurrence and healing of DFU during the follow-up period. A multivariable analysis was performed to assess the association of these outcomes and risk factors.

**Results:**

During the follow-up period, 9.7% of participants died, 12.1% required amputation, 29.2% had a DFU recurrence, and 73.8% healed. Having a caregiver, ischemia or infection were associated with higher mortality risk (hazard ratio [HR]:3.63, 95% confidence interval [CI]:1.05; 12.61, HR: 6.41, 95%CI: 2.25; 18.30, HR: 3.06, 95%CI: 1.05; 8.94, respectively). Diabetic retinopathy was an independent risk factor for amputation events (HR: 3.39, 95%CI: 1.37; 8.39). Increasing age decreased the risk for a DFU recurrence, while having a caregiver increased the risk for this event (HR: 0.97, 95%CI: 0.94; 0.99). The need for a caregiver and infection decreased the probability of DFU healing (HR: 0.57, 95%CI: 0.39; 0.83, HR: 0.64, 95%CI: 0.42; 0.98, respectively). High scores for PEDIS (≥7) or SINBAD (≥3) were associated with an increased risk for DFU recurrence and a lower probability of DFU healing, respectively.

**Conclusions:**

We observed high morbidity among subjects with a new DFU in our primary healthcare facilities. Peripheral arterial disease, infection, and microvascular complications increased the risk of poor clinical outcomes among subjects with DFU.

**Supplementary Information:**

The online version contains supplementary material available at 10.1186/s13047-023-00602-6.

## Background

Diabetic foot and lower limb complications are an important cause of morbidity and mortality among people with diabetes mellitus (DM) [1, 2]. People with diabetic foot ulcers (DFU) require more hospital visits and admissions than those without this complication [3]. Disease-related complications such as DFU can negatively impact the patient's quality of life, as well as increase healthcare costs [1, 2].

Primary healthcare centers are the patient's first contact with the health system in many countries, and its role in the prevention and treatment of chronic conditions such as DM and its complications is fundamental. Therefore, the task of primary health professionals is crucial for the prevention, early detection, and treatment of diabetic foot complications. Increasing the knowledge and awareness of the risk factors that worsen the prognosis of people with DFU at this level of the healthcare system (i.e. primary care) is necessary to act in a more focused, resourceful and decisive way. So far, several studies on the prognosis of the diabetic foot and its associated contributing factors have been carried out in hospital settings, in specialized diabetes clinics and multidisciplinary foot centers [4–11]. However, very few studies have been carried out in the primary care setting [12, 13], and therefore the existing data at this level of the healthcare system are scarce.

The International Working Group of the Diabetic Foot (IWGDF) has published an evidence-based guideline concerning the classification of DFU and the use of classification systems in routine clinical practice [14]. Three types of classifications have been defined: patient-related (morbidity of the patient, e.g., presence of chronic kidney disease), limb-related (peripheral artery disease and loss of protective sensation), and ulcer-related (area, depth, localization, number, and infection). The IWGDF [14] recommends these classifications to facilitate communication among health professionals, for treatment guidance, and for audits of clinical outcomes in healthcare units and populations, but does not recommend their use for prognostic purposes in patients with DM foot ulcers. Indeed, there is a lack of information on the applicability of the different DFU classifications and their prognostic value in primary care.

We carried out a multicenter study in Catalonia (Spain), where the annual incidence of the occurrence of a new DFU during the recruitment period was 0.42% [15].

## Methods

### Aim, design and setting of the study

The aim of the current study was to assess the clinical progression of DFU during a 12-month follow-up period in people with type 2 diabetes (T2DM) with a diagnosis of a new DFU. We conducted a prospective single-cohort observational study in 36 primary care healthcare centers in Catalonia. The health care system in Catalonia is public and universal to all residents. In each primary care center, the health care user (patient) is allocated a medical doctor and a nurse. The primary healthcare centers act as gatekeepers to access other healthcare levels (secondary and tertiary care). In our study, people with T2DM with a new DFU who attended one of the participating centers were included between February 1^st^, 2018 and July 31^st^, 2019. The follow-up period was up to 12 months or until premature discontinuation (death or loss to follow-up). The detailed methodology has been previously published [15].

### Study procedures

During the first month after the inclusion, weekly follow-up visits for each participant were performed. After the first month, in case of active foot ulcers, follow-up visits were scheduled monthly or more frequently when deemed necessary by the treating team. Upon foot ulcer healing, follow-up visits were scheduled every three months or until the end of the follow-up period. All of the study procedures, data collection check-ups and fulfilment of good clinical practice were externally monitored to ensure correct study practices.

### Definition of diabetic foot ulcer, study variables and outcomes

We defined a foot ulcer as a full-thickness lesion below the ankle, regardless of the presence of neuropathy and/or peripheral artery disease. In individuals with more than one ulcer at baseline, the most clinically relevant lesion was selected as the index DFU.

At the inclusion, for each participant in the study, we collected the following demographic and clinical information (variables): socio-demographic characteristics (age, gender, and self-reported ethnicity), toxic habits (smoking, alcohol intake), cardiovascular risk factors and concomitant disease (hypertension, hyperlipidemia, previous history of stroke, ischemic heart disease, peripheral artery disease, and heart failure), and data on diabetes (disease duration, antidiabetic treatment, and previous diagnosis of diabetic microvascular complications such as retinopathy, nephropathy, and peripheral neuropathy), and previous history of amputation or foot ulcers. In addition, for all participants, laboratory parameters were requested at inclusion for HbA1c, lipid profile, kidney function, and clinical parameters such as body mass index (BMI) and blood pressure were measured. We also collected information related to visual acuity, degree of mobility, caregiver access, and any podiatrist visits.

At all study visits, the site researchers also collected information related to the DFU, such as duration, location, the extension of ulcer after surgical debridement (longest diameter multiplied by the second longest diameter of the ulcer), ulcer´s depth (superficial ulcer: loss of superficial substance which does not penetrate beyond the dermis; deep ulcer: loss of substance below the dermis to subcutaneous structures or joint or bone exposure), presence or absence of infection, and infection severity.

The primary study outcomes were: mortality, amputation, recurrence and healing of DFU. Mortality was considered as death for any reason. The amputation event included both minor and major amputations. We defined a minor amputation as any surgical procedure resulting in an amputation of any part of the limb below the foot ankle, and major amputation was defined as any amputation above the foot ankle. DFU recurrence was considered if a new ulcer appeared during the follow-up period once the index ulcer had healed entirely. The healed DFU was defined as a fully epithelialized lesion (with or without amputation).

We used two DFU classification systems: the PEDIS classification [16], which evaluates variables such as Perfusion, Extent, Depth, Infection and Sensation, and the SINBAD classification [17] which includes variables related to the ulcer Site, Ischemia, Neuropathy, Bacterial Infection and Depth based on a scale of 0 to 6. For the PEDIS we used a scoring system (0 to 12) developed by Chuan et al. [18] to facilitate the use of the PEDIS in clinical practice. A PEDIS score of at least seven was considered clinically important, based on the study by Chuan et al. that found that patients with a PEDIS score of at least seven had an increased risk for the composite endpoint of non-healing amputation and death [18]. For the SINBAD classification, we used a scoring system (between 0 and 6) that was created by Ince et al. [17]; we considered a SINBAD score of at least three to be clinically important based on the study by Ince et al. [17] reporting that patients with a score of at least three had a higher risk of non-healing of the ulcer (including amputation and death).

### Statistical methods

Initially we carried out a descriptive analysis of the participants. The qualitative variables were described for number and frequency, and the quantitative variables were summarized by measures of central tendency and dispersion (mean, median, standard deviation, interquartile range). Subsequently we estimated the incidence (cumulative and event rates) for different study outcomes (mortality, amputations, recurrence and healing of DFU) during the follow-up to determine the evolution of the ulcers and the prognosis. Each event rate was estimated as the number of new cases of the event divided by the total person-time at risk during the follow-up period, stratified by type of the DFU (ischemic, neuro-ischemic or neuropathic). After this, we performed a univariate and multivariable proportional hazards analysis to assess the association of the main study outcomes and risk factors, considering the follow-up time. The variables included in the multivariable models were selected based on the clinical criteria. A complete case-analysis was performed. Estimated measures of association were expressed as crude and adjusted hazards ratios (HRs) and their 95% confidence intervals (95%CI). HRs are a measure of how often a particular event happens in one group compared to how often it happens in another group over time. A HR of 1 indicates a lack of an association between the variable (e.g. age) and the event happening (e.g. mortality), a HR greater than 1 indicates an increased risk of the event happening, and a HR below 1 suggests a lower risk of the event happening. To prevent variable collinearity, two different multivariable models were performed for the PEDIS and SIMBAD variables. We used the cox.zph function from the survival package in R (R statistical software) to check the proportional hazards assumption of the Cox models [19]. Furthermore, we included R2 Nagelkerke as an appropriate measure of goodness of fit for each model. Additionally, assumptions of PH Cox model were checked for each parameter [20, 21]. Thereafter, additional reduced models were done removing statistical non-significant variables. For this analysis we used the function cox.zph from {survival} R package (Version 3.3–1). The statistical analyses were performed using R3.6.1 (https://www.r-project.org).

## Results

A total of 256 participants were included. The baseline characteristics are presented in Table 1. Their mean age was 72.2 (12.7) years. Mean diabetes duration was 13.5 (8.1) years, 69.5% were male, 51.6% were treated with insulin, 27.3% had a previous history of DFU, and 8.9% had a previous amputation. Regarding comorbidities, 64.5% of participants had peripheral neuropathy, 65.5% had peripheral artery disease, 32% had diabetic retinopathy, and 57.8% had chronic kidney disease.

**Table 1:** Baseline characteristics of the study participants

Variable	All participants (*n* = 256)
Age, mean (SD), years	72.2 (12.7)
**Gender, *n* (%)**	
Male	178 (69.5)
**Toxic habits, *n* (%)**	
Smokers	50 (19.5)
Former smokers	88 (34.4)
Non-smokers	118 (46.1)
High-risk alcohol intake	14 (5.4)
**Comorbidities, *n* (%)**	
Hypertension	207 (80.9)
Hyperlipidemia	175 (68.4)
Stroke	37 (14.5)
Ischemic heart disease	55 (21.5)
Hearth failure	50 (19.5)
Peripheral artery disease	165 (64.5)
Macrovascular complications	101 (39.5)
Retinopathy	82 (32.0)
Kidney disease	148 (57.8)
Peripheral neuropathy	165 (64.5)
**Clinical variables**	
Diabetes duration, mean (SD), years	13.5 (8.1)
BMI, mean (SD),	29.6 (5.35))
HbA1c, mean (SD), %HbA1c, mean (SD), mmol/mol	7.9 (1.9) 61.3 (14.6)
**Foot characteristics,*n*(%)**	
Previous history of DFU	70 (27.3)
Any previous amputation	23 (8.9)
Any previous major amputation	2 (0.8)
Foot deformities	104 (40.6)
Inadequate footwear	167 (65.2)
Decreased visual acuity	112 (43.8)
Problems with mobility	106 (41.4)
Need of a caregiver	89 (34.8)
At least one podiatrist visit in the previous year	125 (48.8)
**Ulcer site**, *n* (%)	
Toes, plantar	31 (12.1)
Toes, dorsal or interdigital aspect	112 (43.8)
Dorsal or lateral aspect of the foot	53 (20.7)
Plantar forefoot or midfoot	23 (8.9)
Heel	37 (14.5)
Depth of the ulcer*	
Superficial ulcer	197 (77.0)
Deep ulcer	59 (23.0)
Extension of the ulcer**	
≤ 1 cm^2^	144 (56.2)
> 1cm^2^	112 (43.8)
**Ulcer type,***n* (%)	
Neuropathic ulcers	52 (20.3)
Neuro-ischemic ulcers	113 (44.1)
Ischemic ulcers	52 (20.3)
Without peripheral neuropathy or ischemic disease	39 (15.3)
**Infection status,***n* (%)	
No infection	150 (58.6)
Infection	106 (41.4)
Neuropathy and infection	67 (26.1)
PAD and infection	65 (25.3)
**SINBAD classification**	
SINBAD, mean (SD)	2.48 (1.17)
SINBAD < 3	139 (54.3)
SINBAD ≥ 3	117 (45.7)
**PEDIS classification**	
PEDIS, mean (SD)	5.21 (1.87)
PEDIS < 7	191 (74.6%)
PEDIS ≥ 7	65 (25.4%)

*BMI * Body mass index,  *DFU * Diabetic foot ulcer,  *HbA1c * Glycated hemoglobin,  *PAD * Peripheral arterial disease,  *SD * Standard deviation; * superficial ulcer: loss of superficial substance which does not penetrate beyond the dermis; deep ulcer: loss of substance below the dermis to subcutaneous structures or joint or bone exposure

The highest mortality, amputation and DFU recurrence rates were observed among the 113 (44.1%) participants with peripheral neuropathy and peripheral artery disease, while the highest healing rate and shortest time to healing were observed among those without peripheral neuropathy or peripheral artery disease. Supplementary Table 1 shows the events rates and cumulative incidence for the different types of DFU.

In the un-adjusted HR analysis, age, being female, the presence of macrovascular complications, problems with mobility and the need for a caregiver was associated with an increased risk of mortality. Diabetic retinopathy, chronic kidney disease, previous amputation history, or a baseline SINBAD score of 3 points or higher was associated with an increased risk of amputation. Diabetic retinopathy, a personal history of ulcers or amputations, or a baseline PEDIS score of 7 points or higher increased the risk for DFU recurrence, while age and being female decreased the risk for this event. Regarding non-healing, a higher risk was associated with diabetes duration, diabetic retinopathy, chronic kidney disease, a personal history of amputation, frailty variables (mobility or having a caregiver), ischemia, infection and DFU depth. Table 2 shows the results of the unadjusted HR for the different study outcomes and variables.

**Table 2:** Un-adjusted and adjusted hazards ratios for the main study outcomes

Events	Mortality (*n* = 25)		Amputations (*n* = 31)		DFU recurrence (*n* = 75)		DFU healing (*n* = 189)	
Risk factors at baseline	Un Adjusted HR [95%CI]	Adjusted HR [95%CI]	Un Adjusted HR [95%CI]	Adjusted HR [95%CI]	Un Adjusted HR [95%CI]	Adjusted HR [95%CI]	Un Adjusted HR [95%CI]	Adjusted HR [95%CI]
Age (SD)	*1.06 **[1.02;1.10]**	1.00 [0.94; 1.05]	1.01 [0.98;1.04]	1.02 [0.97; 1.06]	*0.98 **[0.97;1.00]**	*0.97 **[0.95; 0.99**]	0.99 [0.98;1.00]	0.98 [0.97; 1.00]
Sex (female), ref.: male	*2.58 **[1.17;5.65]**	2.01 [0.71; 5.72]	0.82 [0.37;1.83]	0.71 [0.24; 2.05]	0.58 [0.33;1.01]	NA	1.01 [0.74;1.38]	1.17 [0.83; 1.64]
Current smoker, ref.: no	0.42 [0.12;1.44]	0.38 [0.07; 2.13]	1.47 [0.63;3.44]	1.77 [0.65; 4.82]	1.48 [0.84;2.60]	1.22 [0.67; 2.22]	1.06 [0.73;1.55]	0.75 [0.50; 1.13]
Any alcohol risk, ref.: no	0.91 [0.40;2.07]	1.41 [0.47; 4.20]	1.97 [0.97;4.01]	2.04 [0.82; 5.05]	1.48 [0.84;2.60]	1.35 [0.98; 1.04]	0.83 [0.61;1.11]	NA
Diabetes duration	1.02 [0.98;1.06]	1.00 [0.95; 1.06]	1.02 [0.98;1.06]	0.98 [0.94; 1.03]	*1.02 **[1.00;1.05]**	1.01 [0.98; 1.04]	*0.98 **[0.96;0.99]**	0.98 [0.97; 1.01]
Hypertension, ref.: no	1.61 [0.48;5.39]	0.78 [0.18; 3.40]	1.12 [0.43;2.93]	0.78 [0.22; 2.86]	0.75 [0.43;1.31]	0.85 [0.42; 1.72]	0.81 [0.57;1.16]	0.98 [0.66; 1.45]
Dislipidemia, ref.: no	0.82 [0.36;1.86]	0.57 [0.22; 1.52]	0.82 [0.39;1.71]	0.48 [0.20; 1.15]	0.74 [0.46;1.18]	0.64 [0.38; 1.07]	0.95 [0.70;1.29]	1.06 [0.77; 1.47]
Macrovascular complications, ref.: no	*3.67 **[1.58;8.52]**	1.08 [0.12; 10.12]	1.19 [0.58;2.43]	1.37 [0.25; 7.63]	1.38 [0.87;2.18]	1.31 [0.56; 3.07]	0.81 [0.60;1.09]	1.00 [0.62 1.60]
Retinopathy, ref.: no	1.17 [0.50;2.72]	1.03 [0.37; 2.86]	*2.97 **[1.46;6.04]**	*3.39 **[1.37; 8.39]**	*1.64 **[1.03;2.61]**	1.17 [0.69; 1.98]	*0.64 **[0.47;0.88]**	0.67 [0.47; 0.96]
Chronic kidney disease (CKD), ref.: no	2.11 [0.96;4.66]	0.79 [0.28; 2.22]	*2.15 **[1.06;4.36]**	1.60 [0.66; 3.86]	1.48 [0.92;2.37]	1.68 [0.96; 2.94]	*0.55 **[0.40;0.77]**	0.73 [0.51; 1.06
History of previous ulcers, ref.: no	0.73 [0.27;1.94]	0.80 [0.23; 2.80]	1.76 [0.85;3.64]	1.28 [0.42; 3.91]	*1.85 **[1.14;2.99]**	NA	0.73 [0.52;1.00]	NA
History of previous amputation, ref.: no	0.46 [0.06;3.44]	0.34 [0.03; 3.47]	*3.08 **[1.32;7.18]**	1.30 [0.36; 4.70]	*2.78 **[1.48;5.19]**	2.62 [1.26; 5.45]	*0.55 **[0.33;0.94]**	0.64 [0.37; 1.11]
HbA1c (%)	0.99 [0.79;1.24]	1.04 [0.79; 1.36]	1.09 [0.90;1.30]	0.96 [0.77; 1.20]	1.03 [0.91;1.16]	0.98 [0.86; 1.12]	0.98 [0.90;1.06]	1.00 [0.91; 1.08]
BMI	1.00 [0.93;1.08]	1.00 [0.91; 1.10]	0.97 [0.90;1.04]	1.01 [0.92; 1.10]	0.98 [0.93;1.02]	0.97 [0.92; 1.02]	1.02 [0.99;1.05]	NA
Decreased visual acuity, ref.: no	1.03 [0.47;2.26]	0.57 [0.22; 1.51]	1.20 [0.59;2.43]	0.63 [0.27; 1.46]	1.16 [0.74;1.83]	0.93 [0.55; 1.57]	0.80 [0.60;1.07]	1.20 [0.87; 1.66]
Problems with mobility, ref.: no	*3.90 **[1.63;9.35]**	2.87 [0.84; 9.84]	1.56 [0.77;3.15]	1.23 [0.47; 3.24]	1.16 [0.74;1.83]	1.16 [0.65; 2.07]	*0.64 **[0.48;0.86**	NA
Need of a caregiver, ref.: no	*5.63 **[2.35;13.5]**	*3.63 **[1.05; 12.61**]	2.00 [0.99;4.06]	2.26 [0.92; 5.55]	1.51 [0.95;2.40]	*1.81 **[1.03; 3.19**]	*0.52 **[0.38;0.72]**	*0.55 [**0.39; 0.79**]
Ischemia, ref.: no	*4.30 **[1.90;9.74]**	*6.41 **[2.25; 18.30**]	*2.20 **[1.08;4.45]**	1.52 [0.59; 3.87]	1.56 [0.98;2.50]	1.47 [0.83; 2.62]	*0.62 **[0.45;0.85]**	0.84 [0.59; 1.18]
Infection, ref.: no	1.07 [0.48;2.38]	*3.06 **[1.05; 8.94**]	*3.33 **[1.57;7.08]**	2.26 [0.69; 7.36]	1.38 [0.88;2.17]	1.50 [0.76; 93]	*0.57 **[0.42;0.77**]	*0.63 [**0.42; 0.96**]
Deep or very deep (Ref: superficial)	0.50 [0.15;1.69]	*0.09 **[0.02; 0.54]**	*2.94 **[1.45;5.97]**	1.95 [0.61; 6.24]	1.49 [0.90;2.45]	0.68 [0.30; 1.54]	*0.55 **[0.38;0.80]**	0.64 [0.38; 1.07]
DFU Extension: > 1 cm, ref. ≤1	1.09 [0.88;1.34]	0.72 [0.26; 1.94]	0.85 [0.69;1.05]	1.84 [0.82; 4.15]	1.07 [0.94;1.21]	NA	1.00 [0.92;1.08]	NA
R2 Nagelkerke		0.217		0.218		0.126		0.234
Global *p*-value**		0.572		0.795		0.146		0.837

*In bold: statistically significant HR; *BMI* Body mass index, *CI* Confidence interval, *DFU* Diabetic foot ulcer, *HbA1c* Glycated hemoglobin, *HR* Hazard ratio, *SD* Standard deviation

** Proportional Hazards Assumption of a Cox Regression test

NA: Estimation not evaluable because it cannot be assumed that it meets the Proportional Hazards Assumption of the COX models

**Fig. 1:**
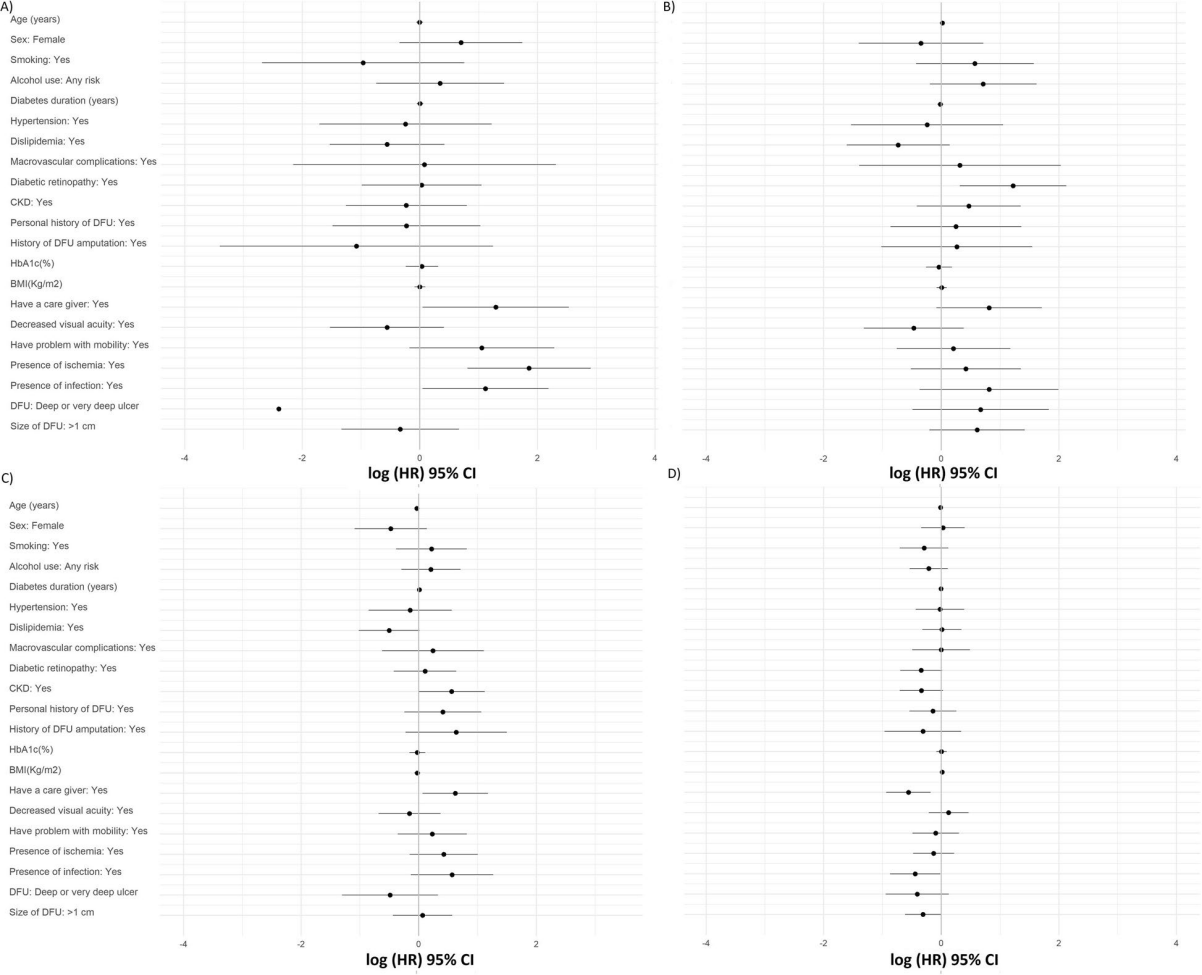
Associations of the main study outcomes with different risk factors, **A**) Mortality **B**) Amputations **C**) DFU recurrence **D**) DFU healing, BMI: body mass index; CKD: chronic kidney disease; CI: confidence intervals; DFU: diabetic foot ulcer; HbA1c: glycated hemoglobin; HR: hazard ratio

**Fig. 2:**
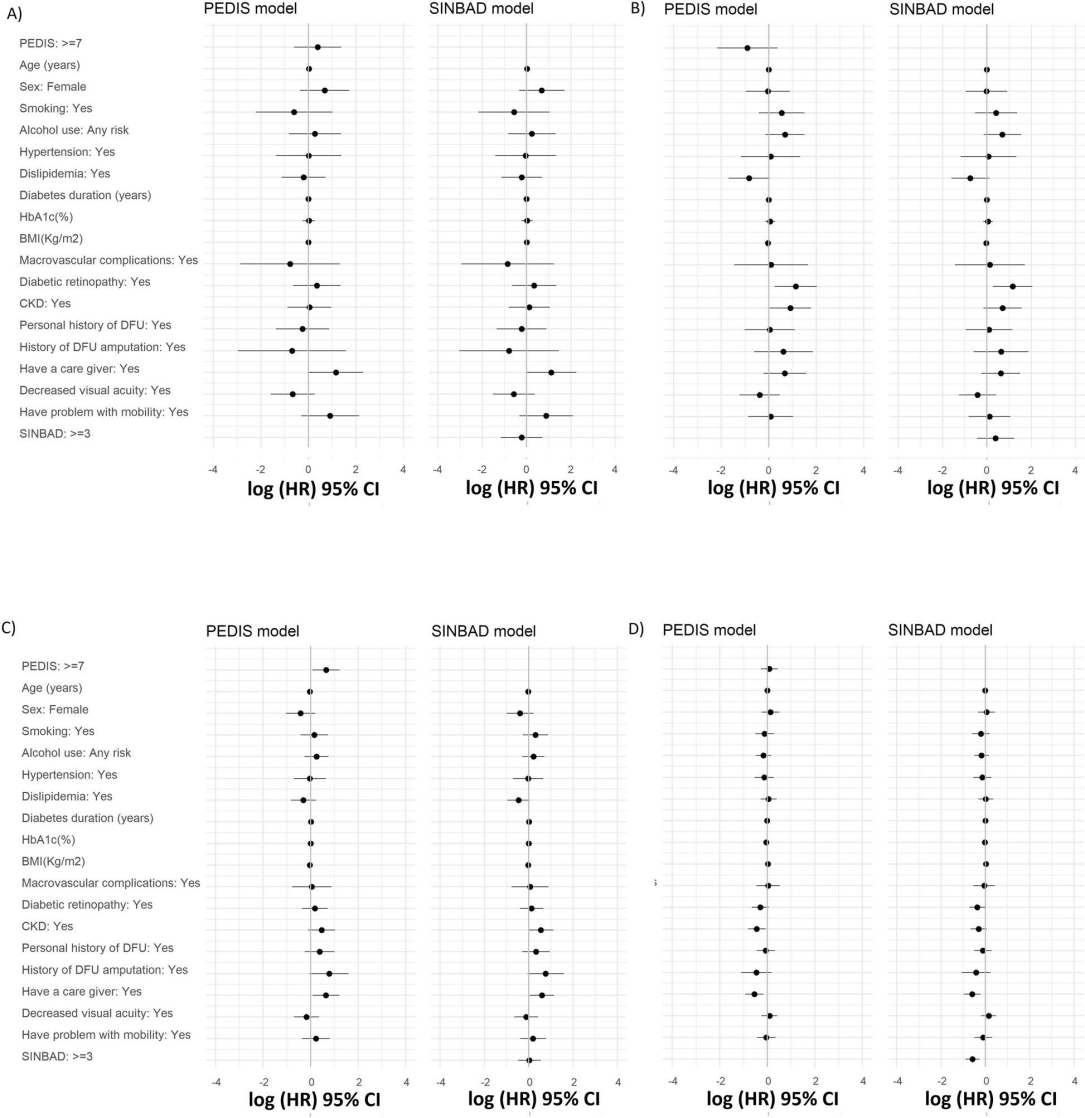
Associations of the main study outcomes with different risk factors in PEDIS and SINBAD models, **A**) Mortality **B**) Amputations **C**) DFU recurrence **D**) DFU healing, BMI: body mass index; CKD: chronic kidney disease; CI: confidence intervals; DFU: diabetic foot ulcer; HbA1c: glycated hemoglobin; HR: hazard ratio

Regarding the multivariable analysis, the need for a caregiver, ischemia or infection were associated with a higher mortality risk. Diabetic retinopathy was an independent risk factor for amputation events. Increasing age decreased the risk for a DFU recurrence, while having a caregiver increased the risk for this event. The need for a caregiver and the presence of infection decreased the probability of healing in the main model. For the additional multivariable models, a PEDIS score of 7 points or higher was only associated with an increased risk of developing a new ulcer, while a SINBAD score of 3 points or higher was only associated with a lower probability of healing. Table 2 and Fig. 1 show the multivariable analysis of risk factors for the main model, while Supplementary Table 2 and 3 and Fig. 2 shows the multivariable analysis for the PEDIS and SINBAD models. Checking the assumptions of PH Cox model, we observed that in the additional reduced models the assumption of PH was not rejected. In these reduced models, similar results were observed for the probability of occurrence of the study events (recurrence and healing of DFU). These additional models are presented in Supplementary Table 4.1–4.12.

## Discussion

Among the 256 participants with T2DM and a new DFU in this multicenter prospective cohort study from different primary care centers in Catalonia, we found a high risk for mortality, amputations and recurrence of a new DFU. So far, similar studies to ours have been carried out in a hospital setting or in multidisciplinary foot-care centers [4–11]. These studies differ from ours, especially in the level of the healthcare system where participants were recruited, and also for the inclusion criteria, the definition of the foot ulcer, and the follow-up time, which make comparisons with our findings difficult. From the studies conducted in primary care settings, similar to ours, Boyko et al. [12] performed a study in US veterans with a follow-up period of 22 years, where all of the participants were males. The study carried out by Muller et al. [13], which assessed the annual incidence of DFU and amputations among T2DM people registered in a database of 4,500 people with different chronic conditions, used a different methodology to ours; around 677 people with diabetes per year were studied between 1993 and 1998, with a reported annual incidence of DFU and amputation of 2.1% and 0.6%, respectively, however, the authors provided very few clinical data, precluding a comparison with participants from our study.

In our cohort, we found a mortality rate of 9.7%. Three hospital-based studies have reported mortality rates in people with DFU with similar follow-up periods to our study [6,8.9]. In the study by Prompers et al. [6] with patients from 14 European hospitals, the authors reported lower mortality rates (6%). However, these people were much younger (mean age 65 years) compared with our participants (mean age 72.2 years). The other two hospital-based studies with similar follow-up periods reported mortality rates much higher than in our study. The study carried out in Germany [8] with type 1 and type 2 diabetic patients showed a mortality rate of 15.4%, while a study carried out in China by Jiang et al. [9] reported mortality rates of 14.4%. The higher prevalence of comorbidities among the people included in the study from Germany and the large number of smokers (43%) in the study from China, could partially explain these high mortality rates. As early as 1990, Apelqvist [22] warned that diabetic patients with a foot ulcer are at high risk of death. A meta-analysis performed by Saluja et al. [23] and by Brownrigg et al. [24] showed that DFU is associated with an increased risk of all-cause mortality compared to those without foot ulceration. In our study, in the adjusted model we did not find that macrovascular events (stroke, ischemic heart disease, heart failure) were associated with an increased risk of mortality in people with DFU as has been reported previously by other authors [5, 11]. In the meta-analysis conducted by Brownrigg et al. [24] the authors observed similar findings to ours regarding cardiovascular events and mortality between people with DFU and without DFU. In our study, we found an increase in mortality among women compared to men, which some authors have previously described and attributed to a greater frailty in women with DFU [25]. We also observed that ischemia and infection increased the risk of death in our multivariable analysis. These variables are well-known risk factors for a poor prognosis in subjects with DFU [26, 27]. The inverse relationship between ulcer depth and mortality may be explained by the difficulty in measuring ulcer depth on many occasions [17, 28].

Regarding amputations, we observed 31 (9.7%) events during the follow-up period, much higher than the annual incidence of this event (0.6%) reported in the cohort study by Muller et al. [13]. In similar hospital-based studies, the incidences of amputation events ranged from 0.05 to 19% [6, 11, 29]. Significant variation exists in the incidence of lower limb amputation even within the same country [30, 31]. We found that the association between amputation and retinopathy was consistent throughout all the models performed. This association was previously described in other studies [32] and indicates the importance of performing ophthalmic examinations in patients with DFU and increasing foot care at the moment of diagnosis of diabetic retinopathy.

Overall, 29.2% of participants in our study experienced a recurrence of a new foot ulcer during the follow-up. There is great variability between the studies regarding this outcome, ranging, for example, from 25% in a study by Muller et al. [13], 32% in a study by Jiang et al. [9] and 43% in a study by Winkley et al. [5]. Age was negatively associated with DFU recurrence, however it is possible that some of the older adults in our study died before the DFU recurrence, and therefore this result should be interpreted with caution. The relationship between the history of ulcers or amputation and recurrence of ulcers found in the univariate analysis disappeared in the multivariable analysis, however this is in contrast to previous studies where both variables (history of ulcers or amputation) have been reported to be poor prognostic factors for DFU recurrence [33].

Regarding DFU healing, we found that in 73.8% of participants the index ulcer healed with or without amputation. Similar results were reported previously by other authors [4, 6, 7, 34]. No relationship was observed between the ulcer's depth, extension and healing, adjusting for different variables, in contrast to what has been reported by other authors [26, 34, 35]. Our analysis highlights that infection is the main variable that interferes with healing. In a study by Prompers et al. [26] no differences were observed for major amputation or healing rate between neuropathic ulcers with and without infection, although infection was a risk factor for minor amputation. In contrast, infection was an independent predictor of poor outcome in patients with peripheral arterial disease, but the prevalence of infection varied markedly between the centers (28–74%)[26].

Needing a caregiver was associated with an increased risk of mortality and with DFU recurrence, while it was negatively associated with ulcer healing. The need for a caregiver may be regarded as a surrogate of frailty. It is well known that frailty is a clinical syndrome associated with dependence and mortality in the older adults, including those with diabetes. Moreover, frailty may be a more powerful prognostic marker than the burden of comorbidity itself [36–38]. This was also the case in the study by Gershater et al. [7], where the authors analyzed a cohort of 2,480 diabetics with a first ulcer and observed that patients with an informal caregiver patient were more likely to have a major amputation or to have died before healing compared to those who did not have a caregiver (odds ratio (OR): 2.16, 95% CI 0.43 – 3.28. *p* < 0.005). The role of informal caregivers remains largely unexplored, and its importance is fundamental in the care of a patient with an ulcer [39].

There are many classifications of people with diabetes mellitus and DFU [14]. The PEDIS classification was designed as an aid for prospective research [14]. Using this classification, Chuan et al. [18] created a scoring system with 364 diabetic foot patients treated in a hospital with a mean follow up of 25 months. The study outcomes were healed DFU and a combination of unhealed DFU, amputation and death. They observed that a PEDIS classification score with a value of at least seven was associated with the worst clinical prognosis of the patients. In our study, a PEDIS score of ≥ 7 was associated with an increased risk of ulcer recurrence during the follow-up period.

Ince et al. [17] conducted a study with diabetic patients with foot ulcers from the UK, Germany, Tanzania and Pakistan to determine the prognostic value of the SINBAD classification score for healing vs no healing, including amputation or death. The authors observed that despite all the differences between countries, ulcers with a SINBAD score of at least 3 had a worse clinical prognosis. In the annual report of the UK National Diabetes Foot Care Audit [40] from 2018, with 19,453 patients with DFU, the SINBAD classification was also used for the same purpose. It was observed that patients with a SINBAD score equal to or greater than 3 were less likely to be alive and ulcer-free at 12 and 24 months. Our study observed that a SINBAD score ≥ 3 was associated with a risk of non-healing during the 12 months follow up period.

This study has some limitations. We have no information on follow-up in 34 patients who discontinued the study. Some limitations, such as possible underreporting, selection bias, and the absence of socioeconomic data, as well as the absence of the prevalence of mental health disorders (depression and anxiety) were previously acknowledged in a prior study [15].

## Conclusions

In conclusion, we observed high morbidity among subjects with a new DFU seen in primary healthcare. As described in previous studies, peripheral arterial disease, infection, and microvascular complications increased the risk of poor clinical outcomes. Further large population-based primary healthcare studies are needed to evaluate the association between different risk factors, especially frailty and severity outcomes of DFUs. Additionally, primary healthcare professionals play a fundamental role in educating people with DM and preventing complications, such as the diabetic foot. Likewise, these professionals must be aware of the importance of ruling out the presence of ischemia and infection in the evaluation and follow-up of DFUs, and to make a prompt referral to secondary/tertiary levels of care when necessary. Coordination between levels of healthcare must be fluid and coordinated. The IWGDF [14] advises using the SINBAD classification in communication between professionals in its latest recommendations. Based on our experience, we believe it can also be a helpful tool for DFU disease course prognosis.

## Supplementary Information

**Additional file 1: Supplementary Table 1.** Event rates for different types of DFU. **Supplementary Table 2. **Adjusted HR for different study outcomes in the PEDIS models. **Supplementary Table 3. **Adjusted HR for different study outcomes in the SINBAD model. **Supplementary Table 4.1 **DFU recurrence as outcome. **Supplementary Table 4.2 Supplementary Table 4.2 **DFU recurrence as outcome. **Supplementary Table 4.3 **DFU healing as outcome. **Supplementary Table 4.4 **DFU healing as outcome. **Supplementary Table 4.5 **PEDIS models for DFU recurrence as outcome. **Supplementary Table 4.6  **PEDIS models for DFU recurrence as outcome. **Supplementary Table 4.7 **PEDIS models for DFU healing as outcome. **Supplementary Table 4.8  **PEDIS models for DFU healing as outcome. **Supplementary Table 4.9 **SINBAD models for DFU recurrence as outcome. **Supplementary Table 4.10 **SINBAD models for DFU recurrence as outcome. **Supplementary Table 4.11 **SINBAD models for DFU healing as outcome. **Supplementary Table 4.12 **SINBAD models for DFU healing as outcome. **Supplementary table 5.** Study site investigators. **Supplementary table 6.** Scientific, clinical and administrative support. 

## Data Availability

The data that support the findings of this study are available from the Fundación redGDPS. Restrictions apply to the availability of these data, due to the nature of the confidential data of the participants of the study.

## Abbreviations

DM: Diabetes mellitus
IWGDF: International Working Group of the Diabetic Foot
DFU: Diabetic foot ulcers
HRs: Hazards ratios
