# Supplementary material for: Prediction of outcomes in subjects with type 2 diabetes and diabetic foot ulcers in Catalonian primary care centers: a multicenter observational study
Source: J Foot Ankle Res. 2023 Feb 28;16:8. doi: 10.1186/s13047-023-00602-6 (PMC9972716; doi:10.1186/s13047-023-00602-6)
Supplement: Supplementary file 1 — Additional file 1: Supplementary Table 1. Event rates for different types of DFU. Supplementary Table 2. Adjusted HR for different study outcomes in the PEDIS models. Supplementary Table 3. Adjusted HR for different study outcomes in the SINBAD model. Supplementary Table 4.1 DFU recurrence as outcome. Supplementary Table 4.2 Supplementary Table 4.2 DFU recurrence as outcome. Supplementary Table 4.3 DFU healing as outcome. Supplementary Table 4.4 DFU healing as outcome. Supplementary Table 4.5 PEDIS models for DFU recurrence as outcome. Supplementary Table 4.6 PEDIS models for DFU recurrence as outcome. Supplementary Table 4.7 PEDIS models for DFU healing as outcome. Supplementary Table 4.8 PEDIS models for DFU healing as outcome. Supplementary Table 4.9 SINBAD models for DFU recurrence as outcome. Supplementary Table 4.10 SINBAD models for DFU recurrence as outcome. Supplementary Table 4.11 SINBAD models for DFU healing as outcome. Supplementary Table 4.12 SINBAD models for DFU healing as outcome. Supplementary table 5. Study site investigators. Supplementary table 6. Scientific, clinical and administrative support. [file 13047_2023_602_MOESM1_ESM.docx]

**ONLINE-ONLY SUPPLEMENTARY MATERIAL**

**These supplemental materials have been provided by the authors to give the readers additional information about the study**

**Prediction of outcomes in subjects with type 2 diabetes and diabetic foot ulcers in Catalonian Primary care centres: a multicenter observational study**

**Authors:** Magdalena Bundó, Bogdan Vlacho, Judit Llussà, Isabel Bobé, Meritxell Aivar, Carmen Ciria , Ana Martínez-Sánchez, Jordi Real, Manel Mata-Cases , Xavier Cos, Montserrat Dòria, Jordi Viade, Josep Franch-Nadal , Dídac Mauricio

| Table of Contents | | Page |
| --- | --- | --- |
| Supplementary table 1 | Event rates for different types of DFU | 2 |
| Supplementary Table 2 | Adjusted HR for different events in the PEDIS model | 3 |
| Supplementary Table 3 | Adjusted HR for different events in the SINBAD model | 5 |
| Supplementary Table 4.1-4.12 | Summary models and proportional hazards assumption for a Cox test | 7 |
| Supplementary Table 5 | Study site investigators | 17 |
| Supplementary Table 6 | Scientific, clinical and administrative support | 18 |

**Supplementary Table 1. Event rates for different types of DFU**

| Type of DFU | Event | Patients | Person/Years | Events | Event rate 10,000 Person -Year | Cumulative incidence (%) |
| --- | --- | --- | --- | --- | --- | --- |
| Ischemic ulcers | Mortality | 52 | 19424 | 6 | 3.09 | 11.54 |
|  | Amputation | 52 | 17039 | 9 | 5.28 | 17.31 |
|  | New foot ulcers | 52 | 15820 | 17 | 10.75 | 32.69 |
|  | Healing | 52 | 6456 | 39 | 60.41 | 75.00 |
| Neuroischemic ulcers | Mortality | 113 | 34283 | 16 | 4.67 | 14.16 |
|  | Amputation | 113 | 29762 | 19 | 6.38 | 16.81 |
|  | New foot ulcers | 113 | 26400 | 37 | 14.02 | 32.74 |
|  | Healing | 113 | 13956 | 76 | 54.46 | 67.26 |
| Neuropathic ulcers | Mortality | 52 | 19700 | 1 | 0.51 | 1.92 |
|  | Amputation | 52 | 19025 | 3 | 1.58 | 5.77 |
|  | New foot ulcers | 52 | 16650 | 14 | 8.41 | 26.92 |
|  | Healing | 52 | 5899 | 44 | 74.59 | 84.61 |
| Without peripheral neuropathy or ischemic disease | Mortality | 39 | 12533 | 2 | 1.60 | 5.13 |
|  | Amputation | 39 | 12533 | 0 | 0.00 | 0.00 |
|  | New foot ulcers | 39 | 10668 | 7 | 6.56 | 17.95 |
|  | Healing | 39 | 2216 | 30 | 135.37 | 76.92 |

**Supplementary Table 2. Adjusted HR for different study outcomes in the PEDIS models**

| Events  Risk factors at baseline | Mortality PEDIS  (n=25) | | Amputations PEDIS  (n=31) | | DFU Recurrence PEDIS  (n=75) | | DFU Healing PEDIS  (n=189 ) | |
| --- | --- | --- | --- | --- | --- | --- | --- | --- |
|  | Un Adjusted HR  [95%CI] | Adjusted  HR  [95%CI] | Un Adjusted HR  [95%CI] | Adjusted  HR  [95%CI] | Un Adjusted HR  [95%CI] | Adjusted  HR  [95%CI] | Un Adjusted HR  [95%CI] | Adjusted  HR  [95%CI] |
| Age (SD) | ***1.06 [1.02;1.10]** | 1.02  [0.97; 1.07] | 1.01  [0.98;1.04] | 0.99  [0.95 ; 1.03] | 0.98  [0.97;1.00] | ***0.97**  [**0.95 ; 0.99**] | 0.99  [0.98;1.00] | 1.00  [0.98 ; 1.01] |
| Sex (female) | ***2.58 [1.17;5.65]** | 2.10  [0.75; 5.87] | 0.82  [0.37;1.83] | 0.96  [0.38 ; 2.44] | 0.58  [0.33;1.01] | 0.65  [0.35 ; 1.21] | 1.01  [0.74;1.38] | 1.14  [0.79 ; 1.65] |
| Current smoker | 0.42  [0.12;1.44] | 0.52  [0.10; 2.63] | 1.47  [0.63;3.44] | 1.72  [0.66 ; 4.48] | 1.48  [0.84;2.60] | 1.17  [0.65 ; 2.09] | 1.06  [0.73;1.55] | 0.89  [0.60 ; 1.31] |
| Any alcohol risk | 0.91  [0.40;2.07] | 1.45  [0.48; 4.41] | 1.97  [0.97;4.01] | 1.98  [0.87 ; 4.51] | 1.48  [0.84;2.60] | 1.28  [0.77 ; 2.12] | 0.83  [0.61;1.11] | 0.85  [0.62 ; 1.17] |
| Diabetes duration | 1.02  [0.98;1.06] | 1.00  [0.95; 1.04] | 1.02  [0.98;1.06] | 1.00  [0.95 ; 1.05] | 1.02  [1.00;1.05] | 1.01  [0.98 ; 1.04] | ***0.98 [0.96;0.99]** | 0.99  [0.97 ; 1.01] |
| Hypertension | 1.61  [0.48;5.39] | 0.99  [0.25; 3.90] | 1.12  [0.43;2.93] | 1.08  [0.31 ; 3.76] | 0.75  [0.43;1.31] | 0.96  [0.49 ; 1.89] | 0.81  [0.57;1.16] | 0.88  [0.58 ; 1.32] |
| Dislipidemia | 0.82  [0.36;1.86] | 0.68  [0.27; 1.74] | 0.82  [0.39;1.71] | 0.44  [0.18 ; 1.04] | 0.74  [0.46;1.18] | 0.74  [0.43 ; 1.26] | 0.95  [0.70;1.29] | 1.05  [0.75 ; 1.47] |
| Macrovascular complications | ***3.67 [1.58;8.52]** | 0.45  [0.06; 3.68] | 1.19  [0.58;2.43] | 1.10  [0.23 ; 5.17] | 1.38  [0.87;2.18] | 1.05  [0.46 ; 2.41] | 0.81  [0.60;1.09] | 1.04  [0.63 ; 1.70] |
| Retinopathy | 1.17  [0.50;2.72] | 1.50  [0.55; 4.09] | ***2.97 [1.46;6.04]** | ***3.10**  [**1.28 ; 7.53**] | ***1.64 [1.03;2.61]** | 1.19  [0.70 ; 2.04] | ***0.64 [0.47;0.88]** | 0.74  [0.52 ; 1.07] |
| Chronic kidney disease (CKD) | 2.11  [0.96;4.66] | 1.25  [0.50; 3.10] | ***2.15 [1.06;4.36]** | ***2.47**  [**1.04 ; 5.88**] | 1.48  [0.92;2.37] | 1.59  [0.90 ; 2.80] | ***0.55 [0.40;0.77]** | ***0.64**  **[0.44 ; 0.93]** |
| Personal history of ulcers | 0.73  [0.27;1.94] | 0.81  [0.26; 2.49] | 1.76  [0.85;3.64] | 1.04  [0.36 ; 3.02] | ***1.85 [1.14;2.99]** | 1.46  [0.78 ; 2.74] | 0.73  [0.52;1.00] | 0.94  [0.63 ; 1.38] |
| Personal history of amputation | 0.46  [0.06;3.44] | 0.48  [0.05; 4.78] | ***3.08 [1.32;7.18]** | 1.86  [0.54 ; 6.44] | ***2.78 [1.48;5.19]** | 2.17  [0.95 ; 4.95] | *0.55 [0.33;0.94] | 0.63  [0.33 ; 1.20] |
| HbA1c (%) | 0.99  [0.79;1.24] | 1.05  [0.80; 1.36] | 1.09  [0.90;1.30] | 1.06  [0.86 ; 1.31] | 1.03  [0.91;1.16] | 1.00  [0.88 ; 1.13] | 0.98  [0.90;1.06] | 0.95  [0.88 ; 1.04] |
| BMI | 1.00  [0.93;1.08] | 1.00  [0.92; 1.09] | 0.97  [0.90;1.04] | 0.97  [0.89 ; 1.05] | 0.98  [0.93;1.02] | 0.96  [0.92 ; 1.02] | 1.02  [0.99;1.05] | 1.03  [1.00 ; 1.06] |
| Decreased visual acuity | 1.03  [0.47;2.26] | 0.53  [0.21; 1.33] | 1.20  [0.59;2.43] | 0.68  [0.29 ; 1.59] | 1.16  [0.74;1.83] | 0.83  [0.49 ; 1.42] | 0.80  [0.60;1.07] | 1.09  [0.78 ; 1.53] |
| Problems with mobility | ***3.90 [1.63;9.35]** | 2.34  [0.69; 7.90] | 1.56  [0.77;3.15] | 1.09  [0.42 ; 2.79] | 1.16  [0.74;1.83] | 1.24  [0.69 ; 2.24] | ***0.64**  **[0.48;0.86** | 0.95  [0.64 ; 1.41] |
| Need of a caregiver | ***5.63 [2.35;13.5]** | ***3.43**  [**1.06; 11.10**] | 2.00  [0.99;4.06] | 1.96  [0.80 ; 4.81] | 1.51  [0.95;2.40] | ***1.90**  [**1.09 ; 3.33**] | ***0.52 [0.38;0.72]** | ***0.58**  [**0.39 ; 0.85**] |
| PEDIS score ≥7 | 1.24  [0.52;2.97] | 1.42  [0.53 ; 3.77] | 0.35  [0.11;1.14] | 0.41  [0.11 ; 1.45] | ***1.66 [1.01;2.72]** | ***1.91**  [**1.08 ; 3.37**] | 1.16  [0.84;1.61] | 1.09  [0.76 ; 1.55] |
| R2 Nagelkerke |  | 0.196 |  | 0.167 |  | 0.143 |  | 0.173 |

***In bold:** statistically significant HR; BMI: body mass index; CKD: chronic kidney disease; CI: confidence intervals; DFU: diabetic foot ulcer; HbA1c: glycated hemoglobin; HR: hazard ratio; SD: standard deviation

**Supplementary Table 3. Adjusted HR for different study outcomes in the SINBAD model**

| Events  Risk factors at baseline | Mortality SINBAD  (n=25) | | Amputations SINBAD  (n=31) | | DFU Recurrence SINBAD  (n=75) | | DFU Healing SINBAD  (n=189 ) | |
| --- | --- | --- | --- | --- | --- | --- | --- | --- |
|  | Un Adjusted HR  [95%CI] | Adjusted  HR  [95%CI] | Un Adjusted HR  [95%CI] | Adjusted  HR  [95%CI] | Un Adjusted HR  [95%CI] | Adjusted  HR  [95%CI] | Un Adjusted HR  [95%CI] | Adjusted  HR  [95%CI] |
| Age(SD) | ***1.06 [1.02;1.10]** | 1.01  **[**0.97; 1.07**]** | 1.01  [0.98;1.04] | 1.00  [0.96 ; 1.04**]** | 0.98  [0.97;1.00] | ***0.97**  [**0.95 ; 0.99]** | 0.99  [0.98;1.00] | 0.99  [0.98 ; 1.00] |
| Sex (female) | ***2.58 [1.17;5.65]** | 2.10  **[**0.75; 5.88**]** | 0.82  [0.37;1.83] | 0.98  [0.38 ; 2.50**]** | 0.58  [0.33;1.01] | 0.68  [0.37 ; 1.24] | 1.01  [0.74;1.38] | 1.05  [0.72 ; 1.53] |
| Current smoker | 0.42  [0.12;1.44] | 0.56  **[**0.11; 2.76**]** | 1.47  [0.63;3.44] | 1.52  [0.59 ; 3.92**]** | 1.48  [0.84;2.60] | 1.36  [0.77 ; 2.40] | 1.06  [0.73;1.55] | 0.81  [0.55 ; 1.20] |
| Any alcohol risk | 0.91  [0.40;2.07] | 1.42  **[**0.4 ; 4.27**]** | 1.97  [0.97;4.01] | 2.02  [0.87 ; 4.67**]** | 1.48  [0.84;2.60] | 1.23  [0.74 ; 2.04] | 0.83  [0.61;1.11] | 0.84  [0.61 ; 1.16] |
| Diabetes duration | 1.02  [0.98;1.06] | 1.00  **[**0.95; 1.05**]** | 1.02  [0.98;1.06] | 0.99  [0.94 ; 1.04**]** | 1.02  [1.00;1.05] | 1.01  [0.98 ; 1.04] | *0.98 [0.96;0.99] | 0.99  [0.97 ; 1.01] |
| Hypertension | 1.61  [0.48;5.39] | 0.94  **[**0.24 ; 3.70**]** | 1.12  [0.43;2.93] | 1.08  [0.31 ; 3.76**]** | 0.75  [0.43;1.31] | 0.97  [0.49 ; 1.90] | 0.81  [0.57;1.16] | 0.87  [0.58 ; 1.30] |
| Dislipidemia | 0.82  [0.36;1.86] | 0.68  **[**0.27 ; 1.71**]** | 0.82  [0.39;1.71] | 0.47  [0.20 ; 1.12**]** | 0.74  [0.46;1.18] | 0.63  [0.38 ; 1.05] | 0.95  [0.70;1.29] | 1.02  [0.73 ; 1.42] |
| Macrovascular complications | ***3.67 [1.58;8.52]** | 0.43  **[**0.05 ; 3.45**]** | 1.19  [0.58;2.43] | 1.15  [0.24 ; 5.50**]** | 1.38  [0.87;2.18] | 1.06  [0.46 ; 2.43] | 0.81  [0.60;1.09] | 0.94  [0.58 ; 1.54] |
| Retinopathy | 1.17  [0.50;2.72] | 1.52  **[**0.56 ; 4.13**]** | ***2.97 [1.46;6.04]** | ***3.22**  [**1.32 ; 7.85]** | ***1.64 [1.03;2.61]** | 1.14  [0.67 ; 1.94] | ***0.64 [0.47;0.88]** | **0.69**  [**0.49 ; 0.99]** |
| Chronic kidney disease (CKD) | 2.11  [0.96;4.66] | 1.33  **[**0.53 ; 3.33**]** | ***2.15 [1.06;4.36]** | 2.03  [0.85 ; 4.82**]** | 1.48  [0.92;2.37] | 1.73  [0.97 ; 3.06] | ***0.55 [0.40;0.77]** | 0.73  [0.51 ; 1.07**]** |
| Personal history of ulcers | 0.73  [0.27;1.94] | 0.85  **[**0.28 ; 2.61**]** | 1.76  [0.85;3.64] | 1.11  [0.39 ; 3.17**]** | ***1.85 [1.14;2.99]** | 1.38  [0.74 ; 2.57] | 0.73  [0.52;1.00] | 0.89  [0.60 ; 1.31**]** |
| Personal history of amputation | 0.46  [0.06;3.44] | 0.43  **[**0.04 ; 4.18**]** | ***3.08 [1.32;7.18]** | 1.90  [0.55 ; 6.54**]** | ***2.78 [1.48;5.19]** | 2.15  [0.95 ; 4.87] | ***0.55 [0.33;0.94]** | 0.66  [0.34 ; 1.25**]** |
| HbA1c (%) | 0.99  [0.79;1.24] | 1.05  **[**0.80 ; 1.38**]** | 1.09  [0.90;1.30] | 1.05  [0.84 ; 1.30**]** | 1.03  [0.91;1.16] | 0.99  [0.87 ; 1.13**]** | 0.98  [0.90;1.06] | 0.97  [0.89 ; 1.06**]** |
| BMI | 1.00  [0.93;1.08] | 1.00  **[**0.92 ; 1.09**]** | 0.97  [0.90;1.04] | 0.97  [0.89 ; 1.06**]** | 0.98  [0.93;1.02] | 0.97  [0.92 ; 1.02**]** | 1.02  [0.99;1.05] | 1.02  [0.99 ; 1.05**]** |
| Decreased visual acuity | 1.03  [0.47;2.26] | 0.57  **[**0.22 ; 1.45**]** | 1.20  [0.59;2.43] | 0.66  [0.28 ; 1.51**]** | 1.16  [0.74;1.83] | 0.89  [0.52 ; 1.51**]** | 0.80  [0.60;1.07] | 1.16  [0.83 ; 1.62**]** |
| Problems with mobility | ***3.90 [1.63;9.35]** | 2.28  **[**0.69 ; 7.57**]** | 1.56  [0.77;3.15] | 1.13  [0.44 ; 2.90**]** | 1.16  [0.74;1.83] | 1.21  [0.68 ; 2.16**]** | ***0.64**  **[0.48;0.86** | 0.91  [0.61 ; 1.35**]** |
| Need of a caregiver | ***5.63 [2.35;13.5]** | ***3.31**  **[1.03 ; 10.65]** | 2.00  [0.99;4.06] | 1.88  [0.78 ; 4.53**]** | 1.51  [0.95;2.40] | ***1.80**  [**1.04 ; 3.14]** | ***0.52 [0.38;0.72]** | ***0.55**  [**0.38 ; 0.80]** |
| SINBAD score ≥3 | **0.72 [0.32;1.63]** | 0.81  **[**0.31 ; 2.08**]** | ***2.29 [1.10;4.78]** | 1.48  [0.64 ; 3.41**]** | 1.36  [0.86;2.14] | 1.03  [0.61 ; 1.72**]** | ***0.60 [0.45;0.80]** | ***0.55**  [**0.40 ; 0.76]** |
| R2 Nagelkerke |  | 0.160 |  | 0.160 |  | 0.126 |  | 0.143 |

***In bold:** statistically significant HR; BMI: body mass index; CKD: chronic kidney disease; CI: confidence intervals; DFU: diabetic foot ulcer; HbA1c: glycated hemoglobin; HR: hazard ratio; SD: standard deviation

**Supplementary Table 4.1 DFU recurrence as outcome**

|  | **Summary models** | | | **Proportional hazards assumption for a Cox test** | | |
| --- | --- | --- | --- | --- | --- | --- |
| *Predictors* | *HR* | *95%CI* | *p* | *chisq* | *df* | *p* |
| Age (Years) | 0.97 | 0.94 – 0.99 | **0.006** | 0.90 | 1 | 0.34 |
| Sex. Female | 0.62 | 0.34 – 1.15 | 0.129 | 4.23 | 1 | 0.04 |
| Smoking. Yes | 1.24 | 0.68 – 2.27 | 0.483 | 1.05 | 1 | 0.31 |
| Alcohol. Any risk | 1.23 | 0.74 – 2.04 | 0.420 | 0.26 | 1 | 0.61 |
| Diabetes duration (Years) | 1.01 | 0.98 – 1.04 | 0.529 | 3.54 | 1 | 0.06 |
| Hypertension | 0.86 | 0.43 – 1.75 | 0.686 | 0.59 | 1 | 0.44 |
| Dislipidemia | 0.60 | 0.36 – 1.01 | 0.056 | 0.35 | 1 | 0.56 |
| Macrovascular complications | 1.27 | 0.53 – 3.03 | 0.585 | 0.68 | 1 | 0.41 |
| Retinopathy | 1.11 | 0.65 – 1.89 | 0.694 | 0.25 | 1 | 0.62 |
| Chronic kidney disease (CKD) | 1.75 | 0.99 – 3.08 | 0.053 | 0.94 | 1 | 0.33 |
| Personal history of ulcers | 1.50 | 0.78 – 2.89 | 0.221 | 4.08 | 1 | 0.04 |
| Personal history of amputation | 1.89 | 0.80 – 4.47 | 0.148 | 0.06 | 1 | 0.80 |
| HbA1c(%) | 0.98 | 0.85 – 1.12 | 0.730 | 0.25 | 1 | 0.62 |
| BMI(Kg/m2) | 0.98 | 0.93 – 1.03 | 0.343 | 1.25 | 1 | 0.26 |
| Need of a caregiver | 1.86 | 1.07 – 3.25 | **0.029** | 2.21 | 1 | 0.14 |
| Decreased visual acuity | 0.85 | 0.50 – 1.45 | 0.557 | 1.17 | 1 | 0.28 |
| Problems with mobility | 1.26 | 0.70 – 2.26 | 0.445 | 0.01 | 1 | 0.92 |
| Ischemia | 1.53 | 0.86 – 2.73 | 0.152 | 0.05 | 1 | 0.83 |
| Infection | 1.76 | 0.87 – 3.55 | 0.113 | 1.44 | 1 | 0.23 |
| Deep or very deep( Ref: superficial) | 0.61 | 0.27 – 1.39 | 0.240 | 0.05 | 1 | 0.82 |
| DFU Extension: >1cm, ref ≤1 | 1.07 | 0.64 – 1.77 | 0.804 | 7.66 | 1 | 0.01 |
| R^2^ Nagelkerke | 0.140 | | | - | - | - |
| GLOBAL |  | | | 35.61 | 21 | 0.02 |

**Supplementary Table 4.2 DFU recurrence as outcome**

|  | **Summary models** | | | **Proportional hazards assumption for a Cox test** | | |
| --- | --- | --- | --- | --- | --- | --- |
| Predictors | HR | 95%CI | p | chisq | df | p |
| Age(Years) | 0.97 | 0.95 – 0.99 | 0.005 | 0.77 | 1 | 0.38 |
| Smoking. Yes | 1.22 | 0.67 – 2.22 | 0.513 | 0.97 | 1 | 0.33 |
| Alcohol. Any risk | 1.35 | 0.83 – 2.21 | 0.227 | 0.17 | 1 | 0.68 |
| Diabetes duration (Years) | 1.01 | 0.98 – 1.04 | 0.426 | 3.78 | 1 | 0.05 |
| Hypertension | 0.85 | 0.42 – 1.72 | 0.644 | 0.57 | 1 | 0.45 |
| Dislipidemia | 0.64 | 0.38 – 1.07 | 0.087 | 0.27 | 1 | 0.60 |
| Macrovascular complications | 1.31 | 0.56 – 3.07 | 0.533 | 0.56 | 1 | 0.45 |
| Retinopathy | 1.17 | 0.69 – 1.98 | 0.565 | 0.19 | 1 | 0.66 |
| Chronic kidney disease (CKD) | 1.68 | 0.96 – 2.94 | 0.072 | 0.94 | 1 | 0.33 |
| Personal history of ulcers | 2.62 | 1.26 – 5.45 | 0.010 | 0.08 | 1 | 0.78 |
| Personal history of amputation | 0.98 | 0.86 – 1.12 | 0.783 | 0.30 | 1 | 0.58 |
| HbA1c (%) | 0.97 | 0.92 – 1.02 | 0.299 | 1.64 | 1 | 0.20 |
| BMI(Kg/m2) | 1.81 | 1.03 – 3.19 | 0.039 | 2.11 | 1 | 0.15 |
| Need of a caregiver | 0.93 | 0.55 – 1.57 | 0.792 | 1.51 | 1 | 0.22 |
| Problems with mobility | 1.16 | 0.65 – 2.07 | 0.621 | 0.01 | 1 | 0.92 |
| Ischemia | 1.47 | 0.83 – 2.62 | 0.186 | 0.00 | 1 | 0.95 |
| Infection | 1.50 | 0.76 – 2.93 | 0.241 | 1.45 | 1 | 0.23 |
| Deep or very deep(Ref: superficial) | 0.68 | 0.30 – 1.54 | 0.357 | 0.05 | 1 | 0.82 |
| Observations | 251 | | |  |  | 24.28 |
| R2 Nagelkerke | 0.126 | | |  |  |  |
| GLOBAL |  | | | 24.28 | 18 | 0.15 |

**Supplementary Table 4.3 DFU healing as outcome**

|  | **Summary models** | | | **Proportional hazards assumption for a Cox test** | | |
| --- | --- | --- | --- | --- | --- | --- |
| *Predictors* | *HR* | *95%CI* | *p* | chisq | df | p |
| Age (Years) | 0.99 | 0.97 – 1.00 | 0.067 | 0.00 | 1 | 0.96 |
| Sex. Female | 1.03 | 0.71 – 1.49 | 0.883 | 1.14 | 1 | 0.29 |
| Smoking. Yes | 0.75 | 0.49 – 1.13 | 0.164 | 0.04 | 1 | 0.84 |
| Alcohol. Any risk | 0.81 | 0.58 – 1.12 | 0.199 | 5.35 | 1 | 0.02 |
| Diabetes duration (Years) | 0.99 | 0.97 – 1.01 | 0.463 | 1.18 | 1 | 0.28 |
| Hypertension | 0.98 | 0.65 – 1.48 | 0.913 | 0.49 | 1 | 0.48 |
| Dyslipidemia | 1.01 | 0.72 – 1.41 | 0.953 | 0.03 | 1 | 0.87 |
| Macrovascular complications | 1.00 | 0.61 – 1.63 | 0.990 | 0.01 | 1 | 0.91 |
| Retinopathy | 0.71 | 0.50 – 1.02 | 0.061 | 0.08 | 1 | 0.77 |
| Chronic kidney disease (CKD) | 0.71 | 0.49 – 1.03 | 0.072 | 1.07 | 1 | 0.30 |
| Personal history of ulcers | 0.87 | 0.58 – 1.29 | 0.483 | 9.28 | 1 | 0.00 |
| Personal history of amputation | 0.73 | 0.38 – 1.41 | 0.349 | 1.26 | 1 | 0.26 |
| HbA1c (%) | 1.00 | 0.92 – 1.10 | 0.939 | 0.00 | 1 | 0.96 |
| BMI(Kg/m2) | 1.01 | 0.98 – 1.05 | 0.342 | 5.46 | 1 | 0.02 |
| Need of a caregiver | 0.57 | 0.39 – 0.83 | **0.004** | 0.63 | 1 | 0.43 |
| Decreased visual acuity | 1.13 | 0.81 – 1.59 | 0.472 | 0.78 | 1 | 0.38 |
| Problems with mobility | 0.91 | 0.61 – 1.35 | 0.631 | 4.92 | 1 | 0.03 |
| Ischemia | 0.88 | 0.62 – 1.24 | 0.455 | 3.32 | 1 | 0.07 |
| Infection | 0.64 | 0.42 – 0.98 | **0.042** | 0.06 | 1 | 0.81 |
| Deep or very deep (Ref: superficial) | 0.66 | 0.39 – 1.13 | 0.134 | 0.97 | 1 | 0.32 |
| DFU Extension: >1cm, ref ≤1 | 0.73 | 0.54 – 1.00 | **0.049** | 5.31 | 1 | 0.02 |
| Observations | 251 | | |  |  |  |
| R^2^ Nagelkerke | 0.255 | | |  |  |  |
| GLOBAL |  | | | 43.49 | 21 | 0.00 |

**Supplementary Table 4.4 DFU healing as outcome**

|  | **Summary models** | | | **Proportional hazards assumption for a Cox test** | | |
| --- | --- | --- | --- | --- | --- | --- |
| *Predictors* | *HR* | *95%CI* | *p* | chisq | df | p |
| Age(Years) | 0.98 | 0.97 – 1.00 | **0.010** | 0.043 | 1 | 0.84 |
| Sex. Female | 1.17 | 0.83 – 1.64 | 0.372 | 1.08 | 1 | 0.29 |
| Smoking. Yes | 0.75 | 0.50 – 1.13 | 0.170 | 0.05 | 1 | 0.82 |
| Diabetes duration (Years) | 0.99 | 0.97 – 1.01 | 0.318 | 1.07 | 1 | 0.30 |
| Hypertension | 0.98 | 0.66 – 1.45 | 0.912 | 0.32 | 1 | 0.57 |
| Dyslipidemia | 1.06 | 0.77 – 1.47 | 0.710 | 0.00 | 1 | 0.97 |
| Macrovascular complications | 0.99 | 0.62 – 1.60 | 0.979 | 0.04 | 1 | 0.85 |
| Retinopathy | 0.67 | 0.47 – 0.96 | **0.027** | 0.13 | 1 | 0.72 |
| Chronic kidney disease (CKD) | 0.73 | 0.51 – 1.06 | 0.099 | 0.81 | 1 | 0.37 |
| Personal history of amputation | 0.64 | 0.37 – 1.11 | 0.110 | 1.12 | 1 | 0.29 |
| HbA1c (%) | 0.99 | 0.91 – 1.08 | 0.784 | 0.01 | 1 | 0.94 |
| Need of a caregiver | 0.55 | 0.39 – 0.79 | **0.001** | 0.59 | 1 | 0.44 |
| Decreased visual acuity | 1.20 | 0.87 – 1.66 | 0.256 | 1.24 | 1 | 0.27 |
| Ischemia | 0.84 | 0.59 – 1.18 | 0.312 | 2.29 | 1 | 0.13 |
| Infection | 0.63 | 0.42 – 0.96 | **0.030** | 0.09 | 1 | 0.76 |
| Deep or very deep(Ref: superficial) | 0.64 | 0.38 – 1.07 | 0.091 | 0.63 | 1 | 0.43 |
| Observations | 251 | | |  |  |  |
| R^2^ Nagelkerke | 0.234 | | |  |  |  |
| GLOBAL |  | | | 10.54 | 16 | 0.84 |

**Supplementary Table 4.5 PEDIS models for DFU recurrence as outcome**

|  | **Summary models** | | | **Proportional hazards assumption for a Cox test** | | |
| --- | --- | --- | --- | --- | --- | --- |
| Predictors | HR | 95%CI | p | chisq | df | p |
| PEDIS score ≥7 | 1.91 | 1.08 – 3.37 | 0.026 | 0.001 | 1 | 0.97 |
| Age(Years) | 0.97 | 0.95 – 0.99 | 0.006 | 0.94 | 1 | 0.33 |
| Sex. Female | 0.65 | 0.35 – 1.21 | 0.174 | 3.94 | 1 | 0.05 |
| Smoking. Yes | 1.17 | 0.65 – 2.09 | 0.602 | 2.14 | 1 | 0.14 |
| Alcohol. Any risk | 1.28 | 0.77 – 2.12 | 0.344 | 0.09 | 1 | 0.76 |
| Hypertension | 0.96 | 0.49 – 1.89 | 0.913 | 0.84 | 1 | 0.36 |
| Dyslipidemia | 0.74 | 0.43 – 1.26 | 0.264 | 0.62 | 1 | 0.43 |
| Diabetes duration(Years) | 1.01 | 0.98 – 1.04 | 0.591 | 3.53 | 1 | 0.06 |
| HbA1c (%) | 1.00 | 0.88 – 1.13 | 0.970 | 0.66 | 1 | 0.41 |
| BMI(Kg/m2) | 0.96 | 0.92 – 1.02 | 0.164 | 1.84 | 1 | 0.18 |
| Macrovascular complications | 1.05 | 0.46 – 2.41 | 0.905 | 0.96 | 1 | 0.33 |
| Retinopathy | 1.19 | 0.70 – 2.04 | 0.514 | 0.19 | 1 | 0.66 |
| Chronic kidney disease (CKD) | 1.59 | 0.90 – 2.80 | 0.113 | 1.23 | 1 | 0.27 |
| Personal history of ulcers | 1.46 | 0.78 – 2.74 | 0.236 | 3.71 | 1 | 0.05 |
| Personal history of amputation | 2.17 | 0.95 – 4.95 | 0.065 | 0.03 | 1 | 0.85 |
| Need of a caregiver | 1.90 | 1.09 – 3.33 | 0.024 | 2.19 | 1 | 0.14 |
| Decreased visual acuity | 0.83 | 0.49 – 1.42 | 0.506 | 0.86 | 1 | 0.35 |
| Problems with mobility | 1.24 | 0.69 – 2.24 | 0.467 | 0.05 | 1 | 0.83 |
| Observations | 251 | | |  |  |  |
| R2 Nagelkerke | 0.143 | | |  |  |  |
| GLOBAL |  | | | 29.23 | 18 | 0.05 |

**Supplementary Table 4.6 PEDIS models for DFU recurrence as outcome**

|  | **Summary models** | | | **Proportional hazards assumption for a Cox test** | | |
| --- | --- | --- | --- | --- | --- | --- |
| Predictors | HR | 95%CI | p | chisq | df | p |
| PEDIS score ≥7 | 1.87 | 1.06 – 3.27 | 0.030 | 0.00 | 1 | 0.98 |
| Age(Years) | 0.97 | 0.95 – 0.99 | 0.003 | 0.77 | 1 | 0.38 |
| Sex. Female | 1.21 | 0.68 – 2.15 | 0.519 | 1.98 | 1 | 0.16 |
| Smoking. Yes | 1.40 | 0.86 – 2.29 | 0.174 | 0.07 | 1 | 0.79 |
| Alcohol. Any risk | 0.93 | 0.47 – 1.83 | 0.830 | 0.84 | 1 | 0.36 |
| Hypertension | 0.76 | 0.45 – 1.28 | 0.298 | 0.46 | 1 | 0.49 |
| Dyslipidemia | 1.01 | 0.98 – 1.04 | 0.566 | 3.53 | 1 | 0.06 |
| HbA1c (%) | 0.99 | 0.87 – 1.13 | 0.892 | 0.55 | 1 | 0.46 |
| BMI(Kg/m2) | 0.96 | 0.92 – 1.01 | 0.130 | 1.89 | 1 | 0.17 |
| Macrovascular complications | 1.10 | 0.48 – 2.52 | 0.828 | 0.79 | 1 | 0.37 |
| Retinopathy | 1.22 | 0.71 – 2.08 | 0.470 | 0.15 | 1 | 0.69 |
| Chronic kidney disease (CKD) | 1.53 | 0.87 – 2.70 | 0.142 | 1.14 | 1 | 0.28 |
| Personal history of ulcers | 1.41 | 0.75 – 2.63 | 0.282 | 3.58 | 1 | 0.06 |
| Personal history of amputation | 2.25 | 0.98 – 5.16 | 0.054 | 0.06 | 1 | 0.81 |
| Need of a caregiver | 1.88 | 1.07 – 3.30 | 0.028 | 2.16 | 1 | 0.14 |
| Decreased visual acuity | 0.90 | 0.53 – 1.51 | 0.686 | 1.13 | 1 | 0.29 |
| Problems with mobility | 1.20 | 0.67 – 2.17 | 0.539 | 0.00 | 1 | 0.93 |
| Observations | 251 | | |  |  |  |
| R2 Nagelkerke | 0.136 | | |  |  |  |
| GLOBAL |  | | | 26.83 | 17 | 0.06 |

**Supplementary Table 4.7 PEDIS models for DFU healing as outcome**

|  | **Summary models** | | | **Proportional hazards assumption for a Cox test** | | |
| --- | --- | --- | --- | --- | --- | --- |
| Predictors | HR | 95%CI | p | chisq | df | p |
| PEDIS score ≥7 | 1.09 | 0.76 – 1.55 | 0.644 | 1.65 | 1 | 0.19 |
| Age(Years) | 1.00 | 0.98 – 1.01 | 0.503 | 0.09 | 1 | 0.77 |
| Sex. Female | 1.14 | 0.79 – 1.65 | 0.491 | 1.54 | 1 | 0.21 |
| Smoking. Yes | 0.89 | 0.60 – 1.31 | 0.547 | 0.04 | 1 | 0.85 |
| Alcohol. Any risk | 0.85 | 0.62 – 1.17 | 0.322 | 4.64 | 1 | 0.03 |
| Hypertension | 0.88 | 0.58 – 1.32 | 0.526 | 1.89 | 1 | 0.17 |
| Dyslipidemia | 1.05 | 0.75 – 1.47 | 0.782 | 0.00 | 1 | 0.97 |
| Diabetes duration (Years) | 0.99 | 0.97 – 1.01 | 0.472 | 1.67 | 1 | 0.19 |
| HbA1c (%) | 0.95 | 0.88 – 1.04 | 0.259 | 0.19 | 1 | 0.67 |
| BMI(Kg/m2) | 1.03 | 1.00 – 1.06 | 0.062 | 3.62 | 1 | 0.06 |
| Macrovascular complications | 1.04 | 0.63 – 1.70 | 0.889 | 0.05 | 1 | 0.82 |
| Retinopathy | 0.74 | 0.52 – 1.07 | 0.108 | 0.00 | 1 | 0.95 |
| Chronic kidney disease (CKD) | 0.64 | 0.44 – 0.93 | 0.019 | 1.26 | 1 | 0.26 |
| Personal history of ulcers | 0.94 | 0.63 – 1.38 | 0.736 | 9.65 | 1 | 0.00 |
| Personal history of amputation | 0.63 | 0.33 – 1.20 | 0.158 | 1.82 | 1 | 0.18 |
| Need of a caregiver | 0.58 | 0.39 – 0.85 | 0.005 | 0.92 | 1 | 0.34 |
| Decreased visual acuity | 1.09 | 0.78 – 1.53 | 0.602 | 1.41 | 1 | 0.23 |
| Problems with mobility | 0.95 | 0.64 – 1.41 | 0.799 | 5.59 | 1 | 0.02 |
| Observations | 251 | | |  |  |  |
| R2 Nagelkerke | 0.173 | | |  |  |  |
| GLOBAL |  | | | 35.44 | 18 | 0.01 |

**Supplementary Table 4.8 PEDIS models for DFU healing as outcome**

|  | **Summary models** | | | **Proportional hazards assumption for a Cox test** | | |
| --- | --- | --- | --- | --- | --- | --- |
| Predictors | HR | 95%CI | p | chisq | df | p |
| PEDIS score ≥7 | 1.09 | 0.77 – 1.55 | 0.620 | 1.61 | 1 | 0.20 |
| Age(Years) | 0.99 | 0.98 – 1.01 | 0.418 | 0.08 | 1 | 0.78 |
| Sex. Female | 1.20 | 0.84 – 1.70 | 0.325 | 1.46 | 1 | 0.22 |
| Smoking. Yes | 0.88 | 0.60 – 1.30 | 0.529 | 0.03 | 1 | 0.87 |
| Hypertension | 0.85 | 0.57 – 1.27 | 0.438 | 1.63 | 1 | 0.20 |
| Dyslipidemia | 1.06 | 0.76 – 1.47 | 0.732 | 0.00 | 1 | 0.97 |
| Diabetes duration (Years) | 0.99 | 0.97 – 1.01 | 0.476 | 1.62 | 1 | 0.20 |
| HbA1c(%) | 0.95 | 0.88 – 1.03 | 0.239 | 0.26 | 1 | 0.61 |
| BMI(Kg/m2) | 1.03 | 1.00 – 1.06 | 0.072 | 3.43 | 1 | 0.06 |
| Macrovascular complications | 1.03 | 0.63 – 1.67 | 0.908 | 0.01 | 1 | 0.91 |
| Retinopathy | 0.73 | 0.51 – 1.05 | 0.086 | 0.01 | 1 | 0.90 |
| Chronic kidney disease (CKD) | 0.63 | 0.44 – 0.92 | 0.016 | 1.24 | 1 | 0.26 |
| Personal history of amputation | 0.57 | 0.33 – 1.00 | 0.049 | 1.68 | 1 | 0.19 |
| Decreased visual acuity | 0.57 | 0.40 – 0.82 | 0.002 | 0.87 | 1 | 0.35 |
| Problems with mobility | 1.11 | 0.81 – 1.54 | 0.513 | 1.52 | 1 | 0.22 |
| Observations | 251 | | |  |  |  |
| R2 Nagelkerke | 0.169 | | |  |  |  |
| GLOBAL |  | | | 17.87 | 15 | 0.27 |

**Supplementary Table 4.9 SINBAD models for DFU recurrence as outcome**

|  | **Summary models** | | | **Proportional hazards assumption for a Cox test** | | |
| --- | --- | --- | --- | --- | --- | --- |
| *Predictors* | *HR* | *95%CI* | *p* | chisq | df | p |
| SINBAD score ≥3 | 1.03 | 0.61 – 1.72 | 0.918 | 0.36 | 1 | 0.55 |
| Age(Years) | 0.97 | 0.95 – 0.99 | **0.008** | 0.73 | 1 | 0.39 |
| Sex. Female | 0.68 | 0.37 – 1.24 | 0.206 | 4.09 | 1 | 0.04 |
| Smoking. Yes | 1.36 | 0.77 – 2.40 | 0.295 | 1.43 | 1 | 0.23 |
| Alcohol. Any risk | 1.23 | 0.74 – 2.04 | 0.418 | 0.12 | 1 | 0.72 |
| Hypertension | 0.97 | 0.49 – 1.90 | 0.922 | 0.74 | 1 | 0.39 |
| Dyslipidemia | 0.63 | 0.38 – 1.05 | 0.079 | 0.48 | 1 | 0.49 |
| Diabetes duration(Years) | 1.01 | 0.98 – 1.04 | 0.467 | 3.86 | 1 | 0.05 |
| HbA1c (%) | 0.99 | 0.87 – 1.13 | 0.928 | 0.41 | 1 | 0.52 |
| BMI(Kg/m2) | 0.97 | 0.92 – 1.02 | 0.246 | 1.56 | 1 | 0.21 |
| Macrovascular complications | 1.06 | 0.46 – 2.43 | 0.892 | 0.59 | 1 | 0.44 |
| Retinopathy | 1.14 | 0.67 – 1.94 | 0.632 | 0.19 | 1 | 0.66 |
| Chronic kidney disease (CKD) | 1.73 | 0.97 – 3.06 | 0.061 | 1.20 | 1 | 0.27 |
| Personal history of ulcers | 1.38 | 0.74 – 2.57 | 0.311 | 4.03 | 1 | 0.04 |
| Personal history of amputation | 2.15 | 0.95 – 4.87 | 0.067 | 0.03 | 1 | 0.86 |
| Need of a caregiver | 1.80 | 1.04 – 3.14 | **0.037** | 2.72 | 1 | 0.09 |
| Decreased visual acuity | 0.89 | 0.52 – 1.51 | 0.661 | 1.27 | 1 | 0.26 |
| Problems with mobility | 1.21 | 0.68 – 2.16 | 0.521 | 0.00 | 1 | 0.97 |
| Observations | 251 | | |  |  |  |
| R^2^ Nagelkerke | 0.126 | | |  |  |  |
| GLOBAL |  | | | 28.97 | 18 | 0.05 |

**Supplementary Table 4.10 SINBAD models for DFU recurrence as outcome**

|  | **Summary models** | | | **Proportional hazards assumption for a Cox test** | | |
| --- | --- | --- | --- | --- | --- | --- |
| *Predictors* | *HR* | *95%CI* | *p* | chisq | df | p |
| SINBAD score ≥3 | 1.07 | 0.64 – 1.79 | 0.793 | 0.33 | 1 | 0.56 |
| Age(Years) | 0.97 | 0.95 – 0.99 | **0.009** | 0.71 | 1 | 0.39 |
| Sex. Female | 1.36 | 0.77 – 2.39 | 0.290 | 1.318 | 1 | 0.25 |
| Smoking. Yes | 1.29 | 0.79 – 2.09 | 0.308 | 0.12 | 1 | 0.73 |
| Alcohol. Any risk | 0.92 | 0.47 – 1.80 | 0.819 | 0.66 | 1 | 0.42 |
| Hypertension | 0.66 | 0.40 – 1.09 | 0.104 | 0.34 | 1 | 0.56 |
| HbA1c(%) | 0.99 | 0.87 – 1.12 | 0.882 | 0.29 | 1 | 0.58 |
| BMI(Kg/m2) | 0.97 | 0.92 – 1.02 | 0.231 | 1.89 | 1 | 0.17 |
| Macrovascular complications | 1.15 | 0.51 – 2.63 | 0.734 | 0.47 | 1 | 0.49 |
| Retinopathy | 1.22 | 0.72 – 2.06 | 0.468 | 0.12 | 1 | 0.73 |
| Chronic kidney disease (CKD) | 1.70 | 0.97 – 2.98 | 0.064 | 1.09 | 1 | 0.29 |
| Personal history of amputation | 2.77 | 1.40 – 5.50 | **0.004** | 0.06 | 1 | 0.80 |
| Need of a caregiver | 1.86 | 1.08 – 3.22 | **0.026** | 2.53 | 1 | 0.11 |
| Decreased visual acuity | 0.94 | 0.55 – 1.59 | 0.807 | 1.62 | 1 | 0.20 |
| Problems with mobility | 1.13 | 0.63 – 2.01 | 0.683 | 0.03 | 1 | 0.85 |
| Observations | 251 | | |  |  |  |
| R^2^ Nagelkerke | 0.114 | | |  |  |  |
| GLOBAL |  | | | 16.46 | 15 | 0.35 |

**Supplementary Table 4.11 SINBAD models for DFU healing as outcome**

|  | **Summary models** | | | **Proportional hazards assumption for a Cox test** | | |
| --- | --- | --- | --- | --- | --- | --- |
| *Predictors* | *HR* | *95%CI* | *p* | chisq | df | p |
| SINBAD score ≥3 | 0.55 | 0.40 – 0.76 | **<0.001** | 1.79 | 1 | 0.18 |
| Age(Years) | 0.99 | 0.98 – 1.00 | 0.182 | 0.01 | 1 | 0.93 |
| Sex. Female | 1.05 | 0.72 – 1.53 | 0.807 | 1.66 | 1 | 0.19 |
| Smoking. Yes | 0.81 | 0.55 – 1.20 | 0.304 | 0.01 | 1 | 0.91 |
| Alcohol. Any risk | 0.84 | 0.61 – 1.16 | 0.285 | 5.84 | 1 | 0.01 |
| Hypertension | 0.87 | 0.58 – 1.30 | 0.499 | 1.24 | 1 | 0.26 |
| Dyslipidemia | 1.02 | 0.73 – 1.42 | 0.927 | 0.06 | 1 | 0.79 |
| Diabetes duration(Years) | 0.99 | 0.97 – 1.01 | 0.538 | 0.95 | 1 | 0.33 |
| HbA1c(%) | 0.97 | 0.89 – 1.06 | 0.511 | 0.11 | 1 | 0.73 |
| BMI(Kg/m2) | 1.02 | 0.99 – 1.05 | 0.112 | 3.33 | 1 | 0.07 |
| Macrovascular complications | 0.94 | 0.58 – 1.54 | 0.813 | 0.03 | 1 | 0.85 |
| Retinopathy | 0.69 | 0.49 – 0.99 | **0.043** | 0.03 | 1 | 0.86 |
| Chronic kidney disease (CKD) | 0.73 | 0.51 – 1.07 | 0.106 | 1.04 | 1 | 0.30 |
| Personal history of ulcers | 0.89 | 0.60 – 1.31 | 0.548 | 8.70 | 1 | 0.00 |
| Personal history of amputation | 0.66 | 0.34 – 1.25 | 0.202 | 1.42 | 1 | 0.23 |
| Need of a caregiver | 0.55 | 0.38 – 0.80 | **0.002** | 0.53 | 1 | 0.47 |
| Decreased visual acuity | 1.16 | 0.83 – 1.62 | 0.386 | 1.19 | 1 | 0.27 |
| Problemswithmobility | 0.91 | 0.61 – 1.35 | 0.627 | 5.01 | 1 | 0.03 |
| Observations | 251 | | |  |  |  |
| R^2^ Nagelkerke | 0.216 | | |  |  |  |
| GLOBAL |  | | | 35.43 | 18 | 0.008 |

**Supplementary Table 4.12 SINBAD models for DFU healing as outcome**

|  | **Summary models** | | | **Proportional hazards assumption for a Cox test** | | |
| --- | --- | --- | --- | --- | --- | --- |
| *Predictors* | *HR* | *95%CI* | *p* | chisq | df | p |
| SINBAD score ≥3 | 0.56 | 0.41 – 0.77 | **<0.001** | 1.94 | 1 | 0.16 |
| Age(Years) | 0.99 | 0.97 – 1.00 | 0.120 | 0.01 | 1 | 0.91 |
| Sex. Female | 1.10 | 0.77 – 1.57 | 0.610 | 1.57 | 1 | 0.21 |
| Smoking. Yes | 0.81 | 0.55 – 1.20 | 0.300 | 0.01 | 1 | 0.89 |
| Hypertension | 0.84 | 0.57 – 1.26 | 0.410 | 1.00 | 1 | 0.32 |
| Dyslipidemia | 1.04 | 0.76 – 1.44 | 0.793 | 0.06 | 1 | 0.81 |
| Diabetes duration(Years) | 0.99 | 0.97 – 1.01 | 0.516 | 0.85 | 1 | 0.35 |
| HbA1c(%) | 0.97 | 0.89 – 1.05 | 0.469 | 0.17 | 1 | 0.67 |
| BMI(Kg/m2) | 1.02 | 0.99 – 1.05 | 0.134 | 3.06 | 1 | 0.08 |
| Macrovascular complications | 0.92 | 0.57 – 1.49 | 0.743 | 0.00 | 1 | 0.94 |
| Retinopathy | 0.68 | 0.48 – 0.97 | **0.031** | 0.06 | 1 | 0.81 |
| Chronic kidney disease (CKD) | 0.73 | 0.50 – 1.06 | 0.094 | 1.09 | 1 | 0.29 |
| Personal history of amputation | 0.56 | 0.32 – 0.98 | **0.041** | 1.29 | 1 | 0.26 |
| Need of a caregiver | 0.54 | 0.38 – 0.77 | **0.001** | 0.48 | 1 | 0.48 |
| Decreased visual acuity | 1.16 | 0.84 – 1.60 | 0.353 | 1.31 | 1 | 0.25 |
| Observations | 251 | | |  |  |  |
| R^2^ Nagelkerke | 0.210 | | |  |  |  |
| GLOBAL |  | | | 16.95 | 15 | 0.32 |

**Supplementary table 5.** Study site investigators

| **Name** | **Surnames** | **Centres** |
| --- | --- | --- |
| Matilde | Garrido Luque | EAP Gòtic |
| Paloma | Prats | EAP Raval Sud |
| Montse | RoderoNuño | EAP Raval Sud |
| Maite | Sanz | EAP Sant Martí |
| Xavier | Peligros Palma | EAP Sant Martí |
| Sandra | Rambla Alsina | EAP Sant Martí |
| Francisco B | Rus Santiago | EAP Sant Martí |
| Mª José‎ | Navarro Hernandez | EAP Sant Martí |
| Francisco | CegriLombando | EAP Sant Martí |
| Isabel | Prieto | EAP PobleNou |
| Carles | Frías | EAP PobleNou |
| Isabel | Bobé | EAP La Mina |
| Dolors | Vila | EAP La Mina |
| Manel | Mata Cases | EAP La Mina |
| Anna | Massana | EAP El Clot |
| Rosa M | Gimbert | EAP Carmel |
| Anna | Martínez | EAP Carmel |
| Eva | Ares Fernàndez | EAP Bon Pastor |
| Verónica | Sierra Peinado | EAP Bon Pastor |
| Meritxell | Aivar | EAP Sants |
| Gemma | RoigRibas | EAP Dr. CarlesRibas |
| Maria Mercedes | Liroz Navarro | EAP Dr. CarlesRibas |
| Gabriel | Cuatrecasas | EAP Sarrià |
| Gloria | Santiago | EAP Sarrià |
| Lola | Martin | EAP Adrià |
| Margarita | Galan Trilla | EAP Adrià |
| Maria Luisa | Marti Aguasca | EAP Adrià |
| Yaiza | Carreras Cabrera | EAP Adrià |
| Amparo | BonetBernabeu | EAP Adrià |
| Pau | Guiu de la Hera | EAP Adrià |
| Silvia | Lorente Gonzalez | EAP Adrià |
| Eduard | Tarragó Sala | EAP Bellvitge |
| Angela | Amorós Molina | EAP Sta. Eulalia Nord |
| Xavier | Calero Ribera | EAP Gornal |
| Rosa Mar | de Miguel | EAP Pubilla Casas |
| Carlos | Gómez Ruiz | EAP Collblanc |
| Irene | Ruiz Tamayo | EAP La Torrassa |
| Míriam | Montserrat Viñals | EAP La Torrassa |
| Judit | Llussà Arboix | EAP Sant Roc |
| Rosa Monica | Blanco Canseco | EAP El Gorg |
| Jordi | Ingla | EAP El Fondo |
| Natalia | CánovasEssard | EAP El Fondo |
| Núria | Casado Pradas | EAP ProgrésRaval |
| Marta | Serra Laguarda | EAP Ca n’Oriac |
| Cristina | Martínez | EAP Ca n’Oriac |
| Mónica | FarràsMasana | EAP Can Trias |
| Marta | Ruiz Toro | EAP Can Trias |
| Mercè | Villaró | EAP Terrassa Sud |
| Mar | Fuentes Fuentes | EAP Terrassa Sud |
| Agnès | Sànchez Almodóvar | EAP Rambla de Terrassa |
| Susana | López Ocaña | EAP Rambla de Terrassa |
| Laura | López Cantero | EAP Rubí 1 |
| Cristina | Soria Segura | EAP Rubí 2 |
| Maite | Pallarès Valverde | EAP Valldoreix |
| Olga | SolbasAvilés | EAP Valldoreix |
| Mireia | Campos Hernández | EAP Valldoreix |
| Ericka | Montalvo Castro | EAP Olesa |
| Monica | Garcia Carrasco | EAP Olesa |
| Mayte | OrtízLupiañez | EAP Sant Andreu de la Barca |
| Mònica | Valverde Castillo | EAP Sant Andreu de la Barca |
| Núria | Saez Rueda | EAP Sant Andreu de la Barca |
| Marta | Sanavia | EAP Penedès Rural |
| Sara | Gonzalez | EAP Les Franqueses |
| Ana Belén | ColladoVicho | EAP Les Franqueses |
| Magda | Bundó | EAP Ronda Prim |
| Mònica | Mestres Massa | EAP Salt |
| Mª Carmen | de Amo Castillo | EAP Salt |
| Xavier | Carbonés | EAP Perelada |
| Judit | Noguera | EAP Perelada |
| Pyrene | Martínez | EAP Perelada |
| Txema | Hernández | EAP Falset |
| Carmen | Ciria | EAP Ponts |
| Maite | Puig Solé | EAP Ponts |
| Angels | LlopMasamunt | EAP La Granadella |
| Neus | Miró Vallvé | EAP Tàrrega |
| Marta | TolosaFortuny | EAP Tàrrega |
| Sandra Maria | Millera | EAP Bordeta-Magranes |
| Teresa | Sola Cinca | EAP Almacelles |
| Anna | Centellas | EAP Navarcles |
| Enric | Hernández Nuet | EAP Corró |
| Mª Jose | Jara Cepeda | EAP Canovelles |
| M. del Carmen | BaenaArjona | EAP Canovelles |
| Mª Carmen | Conde Garcia | EAP Canovelles |

EAP: primary health care team

**Supplementary table 6.** Scientific, clinical and administrative support

| **Name** | **Surnames** | **Position** | **Centres** |
| --- | --- | --- | --- |
| Federico | Vázquez San Miguel | Endocrinologist | Hospital Germans Triasi Pujol |
| Teresa | Mur Martí | Research coordinator for Primary Care | MútuaTerrassa |
| Anna | Moleras | Management Technician | IDIAP Jordi Gol I Gurina |
